# Exploring the role of content and language integrated learning approach in developing transversal skills in university students with respect to the mediating role of emotional intelligence

**DOI:** 10.3389/fpsyg.2022.988494

**Published:** 2022-10-06

**Authors:** Peijun Jiang, Shamim Akhter, Zeinab Azizi, Asma Gheisari, Tribhuwan Kumar

**Affiliations:** ^1^School of International Studies, Hunan Institute of Technology, Hengyang, China; ^2^School of Languages, Civilisation and Philosophy, Universiti Utara Malaysia, Malang, Malaysia; ^3^Teaching English and Linguistics Department, University of Ayatollah Ozma Borujerdi, Borujerd City, Iran; ^4^Department of English, Payame Nour University, Ahvaz, Iran; ^5^College of Science and Humanities at Sulail, Prince Sattam Bin Abdulaziz University, Al Kharj, Saudi Arabia

**Keywords:** content and language integrated learning approach, emotional intelligence, soft skills, transversal skills, communicative competence

## Abstract

Although a range of studies has explored the effects of the content and language integrated learning (CLIL) approach on students’ achievement, its impact on improving university students’ transversal skills (TSs) has remained largely unexplored in Iran. Thus, the present study has a two-fold purpose. First, it aims to investigate the role of the CLIL approach in improving Iranian university students’ TSs. Second, it purports to explore the mediating role of emotional intelligence (EI) in the relationship between the CLIL approach and TSs. For this purpose, a survey design was used wherein a research-made questionnaire was distributed among 123 university students to gather the required data. Findings disclosed that the CLIL approach had a positive role in developing the university students’ TSs. Further, the results indicated that EI positively affected the development of TSs in university students. Moreover, the findings evidenced that EI significantly mediated the relationship between the CLIL approach and the development of the university students’ TSs. The study concludes that applying the CLIL approach involves an entirely new paradigm, offering valuable implications for relevant stakeholders.

## Introduction

Over the last 3 decades, the view of developing transversal skills (TSs) in higher education contexts has received noticeable attention (Ribeiro et al., 2016). According to UNESCO (2013), TSs are defined as the “skills that are typically considered as not specifically related to a particular job, task, academic discipline or area of knowledge and that can be used in a wide variety of situations and work settings (e.g., organizational skills)” (p. 3). As TSs are not specific to particular job duties, sometimes they are called “soft” or “transferrable” skills. The term “transversal” indicates that just like a transversal line in geometry, they cut across different roles and tasks. According to UNESCO (2013), they call fall into six categories, including critical and innovative thinking, intrapersonal skills, interpersonal skills, media and information literacy, global citizenship, and others (i.e., problem-solving, communication, teamwork, and leadership). Such skills are considered highly necessary for reaching the educational objective making students learn them during their study at universities. Moreover, in higher education contexts, explicit learning outcomes have been verified, resulting in the significant improvement of TSs, such as teamwork, communication, and problem-solving (Rakkarnsil and Butsalee, 2022; Veiga, 2022; Zheng, 2022). Additionally, such skills have been linked to the occupation outcomes of higher education students and have been the center of attention for legislators and university officials.

As an example, critical thinking skills are viewed as crucial skills to make correct decisions or solve problems (Liu et al., 2014; Ajmal and Kumar, 2020; Kumar, 2021; Nachbagauer, 2022). Likewise, over the last years, empirical findings have lent support to the need for radical modifications in university syllabuses (Goodwin et al., 2018). They have emphasized the importance of using efficient approaches such as the CLIL approach to assure that students are cultivating a variety of skills, such as collaboration, arbitration, demonstration, and governance (Larraz et al., 2017; Pang et al., 2022; Salomäki et al., 2022).

It is important to note that companies place a premium on personal qualities, such as motivation for working and learning, politeness, dependability, flexibility, punctuality, creativity, and vigor (Larraz et al., 2017; Levine, 2022). Interestingly, the list of TSs favored by companies reveals a preference for individual skills over occupational skills. Companies stress working in a group; the flexibility to organize work autonomously; communication skills; ability to act in the context of the company; recognizing their error; ability to accept criticism; the capacity to act in complex situations; the desire to learn; curiosity; perseverance; politeness; and maintaining a positive attitude (CEDEFOP, 2013, p. 5).

Although TSs have been investigated over the last decade, there are still areas of research that have not been tackled. TSs begin to develop from childhood (Pang et al., 2022; Uygur et al., 2022). According to Carneiro et al. (2007), family and social environment play a significant role in their development. Also, as Ramos et al. (2013) studied, the basics of these skills are established from childhood and they exert a substantial influence on success or failure in all life dimensions. However, Ramos et al. (2013) underscore that the procedures of modeling and improving these skills through the educational platform have yet to be thoroughly investigated due to the existence of multiple research perspectives. However, taking a quick glance at the available literature reveals that no study has explored yet the effects of the CLIL approach on fostering university students’ TSs in the higher education context of Iran. Additionally, the mediating role of emotional intelligence on the effects of the CLIL approach on improving university students’ TSs has remained unexplored. Considering these points, the current research aimed to explore the effects of the CLIL approach on improving university students’ TSs with respect to the role of emotional intelligence. Probably, the findings of the present study can further the pertinent stakeholders’ understanding of the effects of applying the CLIL approach with respect role of emotional intelligence in fostering Iranian university students’ TSs.

## Literature review

### Content language integrated language approach

Content and language integrated learning approach is a dual-focused approach that both language and content receive equal attention (Coyle, 2007; Mehisto et al., 2008; Cenoz et al., 2013; Kuzembayeva et al., 2022). In other words, it is viewed as an educational approach where a foreign language is a medium for teaching curriculum content (Dalton-Puffer, 2011; Vadivel and Beena, 2019; Mahan, 2022). This approach can be implemented as a form of mainstream education at the primary, secondary, and tertiary levels. Lasagabaster and Sierra (2009) verified some of the essential features of the CLIL approach. The first feature is that in the CLIL approach, a foreign language instead of a second language is used, meaning that the students do not encounter the target language outside the classroom. The second feature is that English is the dominant language in the CLIL approach. This is due to the fact that one of the key literacy features around the world is a command of English. The third feature is that the instructors in the CLIL approach are normally non-native speakers of English. They are content experts who aim to instruct content subjects taken from academic/professional disciplines. The fourth feature is that the target language is used to instruct around 50% of the curriculum content. The last feature is that students can attend CLIL classes when they have developed their literacy skills sufficiently in the first language. According to Dalton-Puffer (2011), the CLIL approach can be viewed as “a foreign language enrichment measure packaged into content teaching” (p. 184).

A mass of studies has been conducted on the effects of the CLIL approach on cultivating L2 learning (e.g., Xanthou, 2011; Brevik and Moe, 2012; De Diezmas, 2016). In a study, Xanthou (2011) explored the effects of the CLIL approach on content learning and vocabulary learning among L2 learners. The findings disclosed that the CLIL approach was fruitful to improve the participants’ content learning and vocabulary learning. Besides, Brevik and Moe (2012) examined if the CLIL approach had any effects on Norwegian students’ language achievement. The findings demonstrated that regardless of the student’s proficiency levels, the CLIL approach was effective to boost their language outcomes at the end of 2-year instruction. Additionally, Heras and Lasagabaster (2015) studied the effectiveness of CLIL on L2 learners’ motivation and self-esteem. Also, they inspected the mediating effect of the CLIL approach on gender differences in L2 learning. The results evidenced that the CLIL approach was fruitful to relieve gender differences in motivation. Similarly, they found that the CLIL approach affected positively the participants’ vocabulary learning regardless of their genders. Further, De Diezmas (2016) investigated the effectiveness of the CLIL approach in cultivating L2 learners’ communicative competence. The findings of CLIL learners and non-CLIL learners enrolled in a 4-year primary education evidenced that the participants who were trained based on the principles and procedures of CLIL outperformed the non-CLIL approach learners.

Some reasons have been presented for these results, including better learning conditions owing to authentic lesson materials, the longer period of exposure, extra EFL lessons, the presence of native speakers, and the generally richer linguistic content of the CLIL classes (Dalton-Puffer, 2008; Banegas and del Pozo Beamud, 2022). According to Dalton-Puffer, (2007), the CLIL approach is effective to cultivate L2 learning because it creates a real-life situation in which L2 is used for real-life purposes. This offers valued opportunities for L2 learners to process information more deeply. CLIL environments are found promising for better L2 performers, more motivated, and more academically and linguistically talented (Bruton, 2011; Paran, 2013). In short, as Küppers and Trautmann (2013) suggested, CLIL environments are appropriate for both high-achieving and low-achieving L2 learners.

### Development of transversal skills

The issue of TSs has gained noticeable attention over the last years because nobody can gain independence in learning if they lack these essential skills (Stal and Paliwoda-Pękosz, 2019; Vadivel et al., 2021; Satayev et al., 2022). Previously, different terms such as basic skills, key competencies, employability skills, the 21^st^-century skills, and transversal competencies have been used interchangeably for TSs (Viska project, 2017; Ghadermarzi and Mohamadi, 2022). In a simple definition, the TSs have been viewed as the skills which can be learned in one area or situation and can be used efficiently in other areas and situations (AIC, 2016). According to Whittemore (2018), TSs include collaborative problem solving, digital competencies, learning to learn and continue to learn, initiative and independent thinking, adaptability, resilience, cultural awareness, and expression. In another classification proposed by the United Nations, TSs entail communication skills, teamwork, collaboration skills, planning and organizing, creativity, accountability, commitment to continuous learning, and client orientation. Based on another classification, TSs comprise critical thinking, collaboration skill, communication skill, and creativity and innovation skills (The Ontario Public Service, 2016). Despite the differences among these classifications of TSs, the central point of the skills is that they are the essential skills that are transferable, reusable, and not specifically connected with a job or discipline (Liu et al., 2021; Belchior-Rocha et al., 2022).

In spite of the classification differences, four skills shape the central core of the TSs, including critical thinking, collaboration skills, communication skills, and innovation/creativity (Trilling and Fadel, 2009; Kivunja, 2015). As currently considered the keystone of 21st-century learning, critical thinking is the skill to solve problems (Stanford Encyclopedia of Philosophy, 2018). In other words, critical thinking is the ability to engage in our own thoughts and do reflection to reach a well-informed conclusion. According to Kivunja (2015), critical thinking is a highly curial skill throughout life in modern-day, which is the age of global technologies and economics. As Trilling and Fadel (2009) studied, collaboration skills are viewed as the intrapersonal and interpersonal abilities to collectively make progress toward a common purpose or solve a common problem. Communication skills are defined as the abilities used to give and receive different kinds of information (Maguire and Pitceathly, 2002; Sunday Adebayo et al., 2022; Tonekaboni and Nasiri, 2022). Creative thinking skills are defined as the techniques employed to approach an issue from creative and different perspectives, using the right tools to develop a plan and evaluate it (Basadur et al., 2000; Alnamrouti et al., 2022).

### Emotional intelligence

Emotional intelligence (EI) was introduced and supported by Goleman (1996). According to Goleman (2021), EI can be defined as an individual’s ability “to be motivated and persevere in front of frustration, to govern one’s impulses and to delay personal satisfaction, and to regulate moods and prevent troubles to obscure one’s judgment; to be persistent and have hope” (p. 12). In other words, EI can be viewed as “is the ability to understand, use, and manage your own emotions in positive ways to relieve stress, communicate effectively, empathize with others, overcome challenges, and defuse conflict” (Salovey and Mayer, 2004, p. 12). EI includes different emotions, including anger (e.g., resentment, fury, irritability, indignation, exasperation, and animosity), sorrow (e.g., sadness, being upset, melancholy, loneliness, self-pity, and sulk), fear (e.g., nervousness, anxiety, being afraid, terror, concern, worry, horror, and misunderstanding), joy (e.g., relief, happiness, blessing, pride, pleasure, euphoria, satisfaction, amusement, and contentment), love (e.g., friendship, trust, acceptance, commitment, awe, worship, kindness, and affinity), surprise (e.g., wonder, shock), disgust (e.g., contempt, detestation, revulsion, and repugnance), and shame (e.g., embarrassment, guilt, humiliation, regret, and remorse; Bradberry and Greaves, 2009; Nelson and Low, 2011).

L2 teachers can foster all the dimensions of EI when they are working with learners (Oz, 2015; Li et al., 2021). If they use the appropriate teaching approaches and techniques in the classroom, learners’ EI may raise substantially, leading to promising learning achievements. For this valuable purpose, they are supposed to use teaching approaches and techniques to make students aware of their own feelings, express their own emotions, controlling and regulate their own emotions, and be empathetic (Mayer et al., 2000; Köprü and Ayas, 2020; Khasawneh, 2021).

### Theoretical framework

Modern problem-solving theories posit that language is one of the major media making thinking possible (Jonassen, 2000). It plays a vital role in group discussions, data presentations, and solution presentations (Ortega, 2020; Kumar et al., 2021). As Mitchell et al. (2019) stress, language acquisition occurs when learners are exposed to sufficient comprehensible input. The collaborating negotiation of meaning encourages productivity variations and increases cognizance of the meaning-carrying perspective of the linguistic edifice. As a result, the teaching approaches prioritizing meaning over form make the way for learners to learn and consolidate the linguistic structures (Tavoosy and Jelveh, 2019). One of the teaching approaches predicated on these assumptions is CLIL. It stresses the integration of content and language in learning activities wherein learners need to understand the problematic situation, discuss the model in both spoken and written forms, and produce a clear and understandable solution. Of particular note is that this perspective toward learning is in line with the sociocultural theory (SCT) of learning. From the perspective of SCT, language is regarded as the primary tool for mediating knowledge construction. In a sense, social interactions revolve around language and are perceived as critical media for knowledge and skills construction (Fang et al., 2019). In addition, activity theory (AT) can be considered a suitable theoretical framework for the CLIL approach because it stresses the interaction of the language and content as an instrument to achieve learning outcomes rather than just language or content (San Isidro and Lasagabaster, 2019).

### Research framework

**Figure 1:**
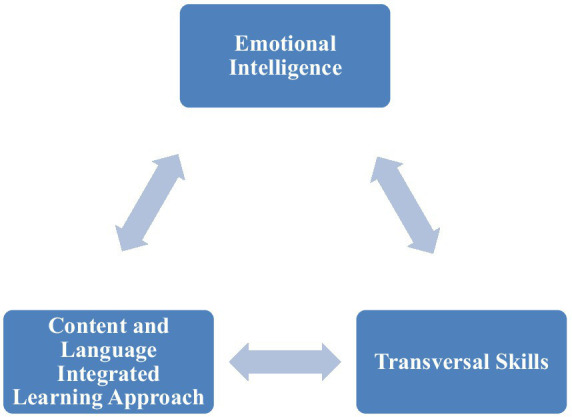
The research framework of the study.

Given the theoretical framework discussed above (Figure 1), the following research hypotheses were put forward:

*H1*. Content and language integrated learning approach play a positive role in developing university students’ transversal skills.

*H2*. Emotional Intelligence positively affects the development of university students’ transversal skills.

*H3*. Emotional intelligence significantly mediates the relationship between the content and language integrated learning approach and the development of the university students’ transversal skills.

## Method of the study

### Research design

This research adopted a quantitative design for collecting the required data. In exact words, a survey design was used wherein a research-made questionnaire was distributed among 123 university students to gather the required data. The primary purpose was to disclose the kind and amount of the relationship between the CLIL approach and the improvement of the university students’ TSs with respect to the mediating role of emotional intelligence. The response rate for the questionnaires is presented in Table 1.

**Table 1:** Response rate for the questionnaires.

Total	Frequencies/Rate
Total distributed questionnaires	150
Total Returned questionnaires	134
Returned and functional questionnaires	123
Returned and omitted questionnaires	09
Response rate	89.3%
Correct response rate	80.2%

### Setting and participants

The present study was conducted at the setting of the University of Ayatollah Ozma Borujerdi, Iran. Representing the major higher education system, state universities in Iran are a type of academic institution to which students need to get a good rank in the national entrance examination (Konkur) to be admitted. Its outstanding features are being free of charge for students, employing high-quality teachers, and following rigorous syllabuses. Using a random sampling method, a total of 150 undergraduate students were selected. As Riazi (2016) noted, the random sampling method provides an equal chance for the individuals in a population to be selected for a study. The underlying reason for selecting the participants was their easy availability to the Iranian researchers in this study. The participants included both males (*n* = 52) and females (*n* = 98) and their ages ranged from 18 to 30 years. They have been studying Applied Linguistics and did not have an opportunity to converse in English outside the walls of the institutes. The participants expressed their consent to take part in the study orally and they were free to withdraw from the study as they wished. It should be noted that the researchers ensured the participants that their responses would be kept confidential and they would be informed about the final results at the end of the study.

### Instruments

To collect the required data, the researchers used a researcher-made questionnaire. They got through the literature meticulously and found that there was not any instrument available to measure the variables in the study. Therefore, in line with the rigorous phases proposed by Crosswell (2005), they designed and developed a questionnaire to measure the variables of interest. In exact words, they followed a three-phase procedure which included planning, construction, and validation. In the first phase, the researchers identified the target group and the purposes of the study. They assured that there was no instrument available in the literature to measure accurately the variables in interest. In the second phase, they identified the objectives of the instrument and developed a table of specifications. They determined the specifications such that the objectives were narrowed down sufficiently and the content areas were identified. They constructed the items and tried to refine them as much as they could. In the third phase, they administered the instrument to 30 university students to measure its reliability and validity. The results showed that the Cronbach’s alpha value was 0.87, indicating that the instrument could readily accept reliability. To measure the validity, the researchers invited two university professors in Applied Linguistics to read and check if the instrument enjoyed a high level of face and content validities. Based on their comments, the researchers modified some of the items in terms of content and language. In general, the questionnaire entailed three sections; the first section consisted of 11 items related to the CLIL approach; the second section included nine questions germane to the development of TSs; the third section contained 12 questions concerning EI. The respondents provided answers on a five-point Likert scale (1 = strongly disagree; 2 = disagree; 3 = neutral; 4 = agree; and 5 = strongly disagree).

### Data collection procedures

The researchers took some distinct steps to conduct the present study. In the first step, they designed and validated the questionnaire. In the second step, they recruited two experts in translation to translate the questionnaire into the participants’ mother tongue (Persian). The primary reason for this was increasing the credibility of the participants’ responses. In the third step, they distributed the questionnaire among the participants. In doing so, they got the participants’ phone numbers from the managers of the institutes and contacted them. They introduced themselves and explained the objectives of the study and asked if they were willing to take part in it. In general, 150 learners agreed to participate in the study and sent their emails and their WhatsApp IDs to the researchers. The researchers sent a digital format of the questionnaire to the participants. In total, 134 university students completed the questionnaire and 123 cases were filled out appropriately. It should be noted that the participants’ responses were stored in a digital database.

## Results

The Partial Least Squares Structural Equation Modeling (PLS-SEM) method was used to measure the relationship between the dependent and independent variables. As Pallant (2020) suggested, PLS-SEM is considered for structural equation modeling by researchers to estimate the complex cause–effect relationships in path models with latent variables. TSs were taken as the dependent variable, and CLIL approach was viewed as the independent variable, and EI was considered as the mediating factor between them. After collecting the raw data with the help of the probability sampling method, all the sections of the questionnaire were coded and keyed into SPSS, version 18. Afterward, a reliability test was conducted, and the results are presented in Table 2.

**Table 2:** Results of the reliability rest.

Construct	No. of items	Cronbach’s Alpha
Transversal Skills	9	0.718
Content and Language Integrated Learning	11	0.825
Emotional Intelligence	12	0.783

### Measurement model

The next stage in the analysis was to examine if the construct items contributed to the hypothesized model as a whole, and for this purpose, the measurement model was examined. Evaluation of the measurement model includes convergent validity assessment (extracted average variance, outer loading, and composite reliability). It is essential to establish the validity of any research to get more accurate and reliable results. Discriminating validity and the extracted average variance (AVE) are two validity criteria evaluations (Hair et al., 2018). Both the dependent variable (TSs) and the independent variable (CLIL approach) were examined in the measurement model. The model was used to measure the construct and its validity. A confirmatory factor analysis (CFA) was performed using PLS-SEM to compute the discriminant validity of the hypotheses. Table 3 reports that all premises meet this condition, hence signifying the discriminant validity of the model.

**Table 3:** Results of the discriminant validity.

	CLIL	EI	TVS
CLIL	0.84		
EI	0.57	0.82	
TVS	0.58	0.68	0.83

**Figure 2:**
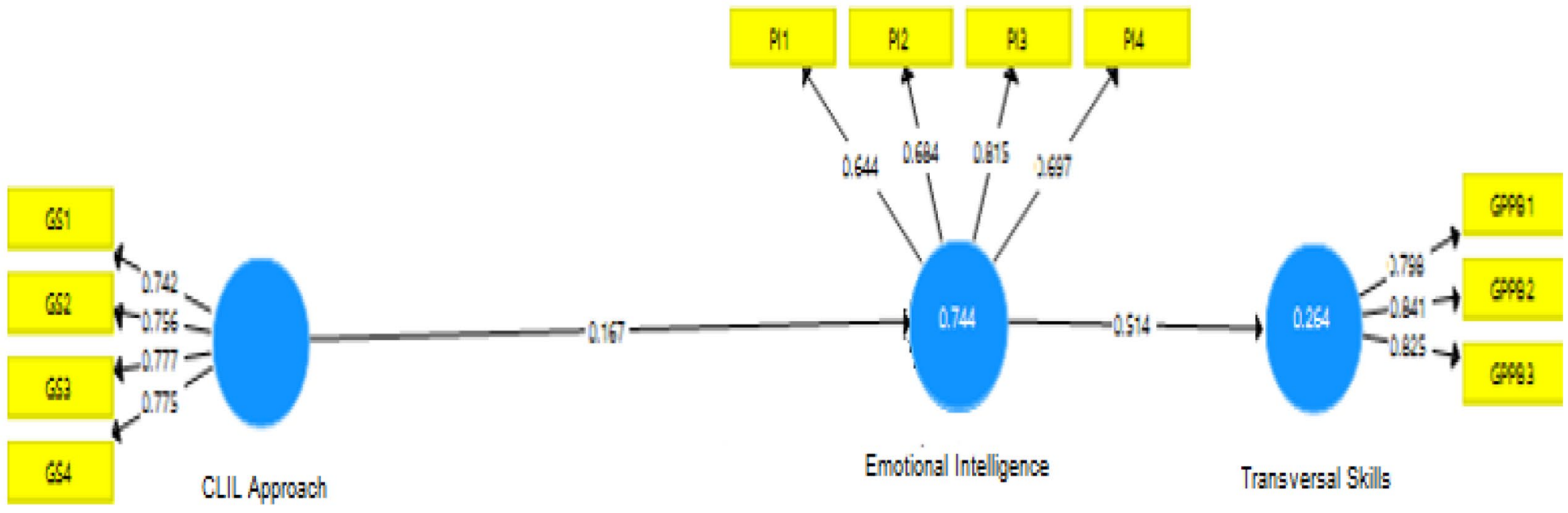
The measurement model.

By proving the construct validity of the measurement model, it was assumed that the questionnaire results were valid and reliable (Figure 2).

### Structural model

**Figure 3:**
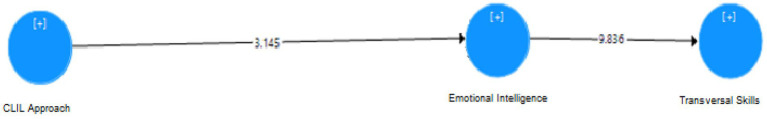
The structural model.

After ensuring that the measurement model was reliable and valid, the structural model was evaluated in the succeeding step. The structural model or inner model assessed the predictive abilities and the relationship between latent constructs. Analysis of the model’s predictive ability and interactions between different components are part of this stage (Hair et al., 2018). The structural model measured the predictive skills and the relationship between latent constructs. After considering multi-collinearity in the previous step, it was proven that there is no multi-collinearity issue among the exogenous variables (Figure 3).

### Coefficient of determination (*R*^2^)

This research employed Smart PLS to evaluate the structural model, including coefficients for the right direction and values for *R*^2^. The path coefficients are standardized regression coefficients used to explain the direction of variables’ relationships. *R*^2^ values represent the structural explanatory power and represent the proportion of variation in endogenous variables (Hsiao and Bomhold, 2013). With higher descriptive power levels, the *R*^2^ ranges from 0 to 1. *R*^2^ values of 0.75, 0.50, and 0.25 are used as guidelines for substance, moderation, and weakness, respectively (Hair et al., 2019).

### Effect size (ƒ^2^)

The impact size indicates the relative effect on a specific exogenous latent variable’s endogenous latent variable with R-square adjustments (Avkiran, 2018). The external structure is critical in explaining the endogenous structure as a static measurement. The effect size (ƒ^2^) is utilized in analysis to assess whether the removed construct has a substantial effect on the endogenous constructs, and the effect size may be calculated by comparing the rise in *R*^2^ to the percentage of the variance of the remaining unexplained endogenous latent variable. As a rule of thumb, 0.02–0.14, 0.15–0.34, and greater than 0.35 are defined by Cohen (2013) as minimal, moderate, and high effects, respectively.

### Path coefficients

Partial Least Squares Structural Equation Modeling uses the path coefficient to evaluate the power and significance of the latent construct’s hypothesized relations. Estimates are derived with a coefficient closer to +1, indicating a strong positive link and a coefficient closer to −1, showing a strong negative relationship and structural model associations with constant values between −1 and + 1.

### Direct and mediating relationships

Assessment of the structural model started with the exploration of the direct relation between the CLIL approach and TSs. The PLS-SEM algorithm was employed to inspect the size of path coefficients, while the significance of the relationship between variables was observed using PLS-SEM bootstrapping technique. The first model consisted of studying the direct relationship among variables H1 and H2 (Table 4). After that, the second model introduced a mediating variable, and the relationship between the independent and mediator variable H3 was examined (Table 5).

**Table 4:** Results of hypotheses testing (Direct relationships).

Theorized Path	Path coefficient	Standard Error	*t* value	*p* value	Decision
H1: CLIL - > TVS	0.348	0.116	2.703	0.007	Accepted
H2: EI - > TVS	0.878	0.122	6.623	0.000	Accepted

**Table 5:** Results of mediating tests.

	Hypothesized path	Path coefficient	Standard Error (STERR)	*t* value	*p* value	Decision
H3	CLIL - > EI- > TVS	0.305	0.108	2.343	0.02^*^	Supported

* *p* < 0.05.

As shown in Table 4, the claim of the hypotheses H1 (e.g., CLIL approach has a role in developing the university students’ TSs) and the claim of H2 (e.g., EI has a role in developing the university students’ TSs) were supported. Similarly, as presented in Table 5, the claim of H3 (e.g., EI mediates the relationship between the CLIL approach and TSs) was also supported.

## Discussion

As noted above, this study purported to explore the role of the CLIL approach in developing university students’ TSs in the higher education in Iran. Additionally, it aimed to disclose the mediating role of EI on the relationship between the CLIL approach and the university students’ TSs. For these purposes, three hypotheses were formulated. The first hypothesis posited that the CLIL approach plays a positive role in developing university students’ TSs. This hypothesis was accepted because, as reported above, the Path coefficient for CLIL > TVS was 0.348, while the *t* value was 2.703 in the structural model’s results. The hypothesis had a *p* of 0.007, more significant than the value of *t* > 1.96, which had a *p* < 0.05. The second hypothesis stated that EI positively affects the development of TSs in university students. This hypothesis was also accepted because, as reported above, the Path coefficient for EI > TVS was 0.878, and the *t* value was 6.623; as evidenced by the results of the structural models. The hypothesis had a value of *p* was 0.000, which was more than the threshold value of *t* > 1.96, which had *p* < 0.05. The third hypothesis stated that EI significantly mediates the relationship between the CLIL approach and the development of the university students’ TSs. This hypothesis was accepted because, as reported above, the Path coefficient for CLIL > EI > TVS was 0.305, while the *t* value was 2.343 in the structural model’s results. The hypothesis had a value of *p* of 0.002, more significant than the threshold value of *t* > 1.96 (*p* < 0.05).

The findings of the study are in line with those of Xanthou (2011), reporting that CLIL was fruitful to improve the participants’ content learning and vocabulary learning. Besides, the results of the study are consistent with those of Brevik and Moe (2012), revealing that regardless of the proficiency levels in students, the CLIL approach was effective to boost their language outcomes at the end of 2-year instruction. Additionally, the findings of this research lend support to the results of Heras and Lasagabaster (2015). They found that the CLIL approach was fruitful to relieve gender differences in motivation. Similarly, they found that the CLIL approach affected positively the participants’ vocabulary learning regardless of their genders. Moreover, in agreement with the results of this study, De Diezmas (2016) found that the participants who were trained based on the principles and procedures of CLIL outperformed the non-CLIL learners.

Based on the findings, it can be argued that the university students’ TSs could develop substantially because they were instructed based on the principles and procedures of the CLIL approach. In other words, the results of the study can be discussed from this perspective that when the university students’ attention was drawn to both the content and language simultaneously, they might have had better opportunities to foster their TSs, such as critical skills, collaborative skills, communication skills, and creative skills. In addition, aligned with the findings of the study, it can be argued that IE had a substantial mediating role in the relationship between the CLIL approach and the development of the TSs. The results are in line with those of the previous studies (e.g., Jonassen and Rohrer-Murphy, 1999; Kolb et al., 2001; Coupal, 2004; Asal and Kratoville, 2013; Olusegun and Bada, 2015), reporting that EI is an influential factor in developing students’ learning skills. In line with the results of the study, it may be argued that a high level of EI increased the students’ chances to develop TSs, which, accordingly, might have led to more promising learning achievements among university students. Along with Nagahi et al. (2020), it may be argued that the CLIL approach with its didactical procedures, using active methods, and integration awareness training in learning, integrating the content and language might have offered ample opportunities for university students to develop TSs.

Furthermore, another line of discussion for the findings may be ascribed to the fact that because the attention of university students was directed toward content provision and acquisition rather than chances for general competence, they might have been in a better position to reconsider their subject’s objectives and learning goals to attain them. The findings of the study are congruent with those of Macianskiene (2016), revealing that the CLIL approach was found to be effective in developing TSs among university students in Lithuania. The findings were ascribed to this reason that the CLIL approach could offer opportunities to study meaningful content in English, allow the participants to join authentic communication, let them compare the newly-acquired information with that already acquired, and set the scene for them to use English to critically think to solve their learning problems. Additionally, aligned with Macianskiene (2016), it may be argued that since the CLIL approach might create an educational environment in which the university students could develop collaborative skills and communication skills, their cognitive skills might have developed, their creativity might have raised, conditions for developing their personality might have set, their cultural awareness might have cultivated, and their expression might have fostered leading to the fostering of TSs.

To discuss the findings of the study, it may be argued that as the CLIL approach was a cohesive method to increase the exposure to the target language and contents without the need to double the amount of time they spent in conventional classes, it might have been found useful to foster the participants’ TSs. In other words, as university students might learn new concepts, they might have increased their time spent in English learning (Lung-Guang, 2019). Moreover, another reason for the results of this study may be attributed to the fact that instead of forcing the university students to focus on the forms of language, the CLIL approach might have directed the university students’ attention to the meanings. That is, the university students’ use of English might have become more contextualized, genuine, and meaningful due to the instruction on topic matters in English. Additionally, as the CLIL approach could provide real-world tasks, the English language might have become natural and much more relevant for the university students, increasing their motivation to think critically, seek innovative ways of learning, and find more chances to communicate and collaborate with others (Injadat et al., 2020; Lyken-Segosebe et al., 2020). The next line of discussion for the results of the study is that as the development of TSs might have been complex, the university student might have been obliged to pay more attention to the learning motivation, formative evaluation, and interactive learning processes (El Ajraoui et al., 2019). These all useful conditions might have been created properly by implementing the CLIL approach.

The other reason for the findings of the study may be lined with this view that the CLIL approach might cope with the lack of relevance of L2 teachings, such as vocabulary and grammar. In a sense, it may be argued that the participants’ motivation might have increased because the CLIL approach might create a naturalistic learning setting. This environment might have motivated the participants to use language, learn content, foster cognitive development, develop communication skills, learn the culture, and encourage their creativity (Harrop, 2012). Put it in a nutshell, the implementation of the CLIL approach might serve the university students’ needs and wants.

To discuss the other part of the findings, which reveal that EI had an effective mediating role in the development of the university students’ TSs, it may be argued that the university students with higher EI levels might have been more tolerant in learning environments that looked like real-life situations wherein English was put to real use. This, in turn, might have offered opportunities for university students to process and internalize the information more efficiently (Dalton-Puffer, 2007). Thus, it is reasonable to argue that higher the EI, the more critical, innovative, and efficient communicator, and collaborator. Besides, the results of the study may be argued from this view that the participants with a higher level of motivation might have had the capabilities in inspiring themselves to develop their TSs (Trilling and Fadel, 2009). In other words, it may be argued that because the students with high levels of EI could manage their motivation efficiently, might have been exposed to more activities to foster TSs. The final reason for the findings may be ascribed to this view that the university students who enjoyed high levels of EI might have been more consistent. This consistency might have been related not only to the classroom environment, but it might have emphasized the significance of lifelong learning, leading to the development of further development and application of TSs throughout life.

## Conclusion and implications

As noted above, the findings disclosed that the CLIL approach played a positive role in developing the TSs of university students. Further, the results indicated that EI positively affected the development of university students’ TSs. Moreover, the findings evidenced that EI significantly mediated the relationship between the CLIL approach and the development of the university students’ TSs. Based on the findings of the study, it may be concluded that the CLIL approach can be implemented to foster TSs which occupy the central place in the lifelong context. The findings gave the conclusion that EI can be cultivated so as to play a positive role in improving TSs. TSs, regardless of job and discipline, are characterized by relevance, necessity, reusability, and transformation (Larraz et al., 2017).

In line with the results of the study, a range of implications are presented for different stakeholders. The first implication is for educational policy-makers to reconsider the positive role of the CLIL approach and EI in fostering students’ TSs. As a result, they should take urgent steps so that this approach and EI can be accommodated in the education system. The second implication is for teacher trainers. They are required to hold pre-service and in-service training workshops to make L2 teachers familiar with the principles and procedures of the CLIL approach and allow them to gain a comprehensive understanding of the significance of EI in second language education. In these training workshops, they should be educated on the theoretical tenets and the practical activities such that they could implement the CLIL approach and EI efficiently in their classes. The third implication is for material developers. For this, they are suggested to design and develop the educational materials in line with the principles of the CLIL approach and EI such that they can cultivate the students’ TSs substantially. The fourth implication is for language teachers. If they are to develop students’ TSs, they need to implement the CLIL approach in their classes with respect to using activities that raise students’ EI. The fifth implication of the findings is for language learners who aim to improve their TSs. To do so, they are supposed to join courses that are run based on the principles and procedures of the CLIL approach, and EI is given enough attention. The last implication is for L2 learners’ parents. They can register their children in courses that integrate the learning of language and content together.

Due to time and resource constraints, the current research suffered from some limitations. These limitations might pave the way for further studies in the future. First, as this study relied on the data collected from undergraduate students in Iran; thus, the results are only applicable to the Iranian higher education contexts, future research can replicate the present study in other parts of the world to generalize the credibility of the findings. Second, as the collected data were quantitative, further studies can be conducted using qualitative designs to disclose how the CLIL approach improves students’ TSs. Third, since the present study was cross-sectional, interested researchers can carry out longitudinal studies to disclose how the CLIL approach affects the development of TSs over a period of time. Fourth, as the current research was conducted in the setting of higher education, interested researchers can further explore the effects of the CLIL approach on improving high school students’ TSs. Last but not least, because the current study focused on the impact of the CLIL approach on the improvement of TSs with respect to the mediating role of EI, future research can expand the spectrum by including more variables in the research analysis.

## Data availability statement

The original contributions presented in the study are included in the article/supplementary material, further inquiries can be directed to the corresponding author.

## Author contributions

All authors listed have made a substantial, direct, and intellectual contribution to the work and approved it for publication.

## Funding

This work was funded by science research of Hunan Education Department (No. 21C0803).

## Conflict of interest

The authors declare that the research was conducted in the absence of any commercial or financial relationships that could be construed as a potential conflict of interest.

## Publisher’s note

All claims expressed in this article are solely those of the authors and do not necessarily represent those of their affiliated organizations, or those of the publisher, the editors and the reviewers. Any product that may be evaluated in this article, or claim that may be made by its manufacturer, is not guaranteed or endorsed by the publisher.
